# Psychosocial work stress and parent-child bonding during the COVID-19 pandemic: clarifying the role of parental symptoms of depression and aggressiveness

**DOI:** 10.1186/s12889-022-14759-5

**Published:** 2023-01-16

**Authors:** Mirjam I. Koerber, Judith T. Mack, Lara Seefeld, Marie Kopp, Victoria Weise, Karla Romero Starke, Susan Garthus-Niegel

**Affiliations:** 1 Institute and Policlinic of Occupational and Social Medicine, Faculty of Medicine, Technische Universität Dresden, Fetscherstr. 74, 01307 Dresden, Germany; 2 Department of Psychotherapy and Psychosomatic Medicine, Faculty of Medicine TU Dresden, Dresden, Germany; 3 Institute for Systems Medicine (ISM), Faculty of Medicine, Medical School Hamburg, Am Kaiserkai 1, 20457 Hamburg, Germany; 4 Department of Childhood and Families, Norwegian Institute of Public Health, N-0213 Oslo, Norway

**Keywords:** DREAM study, COVID-19, Psychosocial work stress, Parent-child bonding, Depression, Aggressiveness

## Abstract

**Background:**

Parental work stress and impaired mental health seem to have intensified during the current COVID-19 pandemic. Both can have a negative impact on parent-child bonding: psychosocial work stress in the course of a spillover effect from work to family and symptoms of impaired mental health as part of a crossover effect from parent to child. This potentially affects the child’s development in the long term.

**Method:**

This cross-sectional study examined the relationship between psychosocial work stress and parent-child bonding during the early COVID-19 pandemic (May–June 2020). Symptoms of depression and aggressiveness were considered as mediators of this relationship. The sample consisted of employees in Eastern Germany (*n* = 380; 42.9% mothers, 57.1% fathers), aged 24–55 years, with children aged 0–36 months.

**Results:**

In the total sample, an association was only found after adjusting for potential confounders, indicating that higher psychosocial work stress is associated with weaker bonding between the parent and child (β = 0.148, *p* = .017, 95% CI [0.566, 5.614]). The separate analyses for mothers and fathers did not reveal a statistically significant relationship between psychosocial work stress and parent-child bonding. In the total sample, the higher the psychosocial work stress was, the higher were the parental symptoms of depression (β = 0.372, *p* < .001, 95% CI [3.417, 5.696]) and aggressiveness β = 0.254, *p* < .001, 95% CI [1.008, 3.208]). The mental health symptoms in turn were related to weaker parent-child bonding (symptoms of depression β = 0.320, *p* < .001, 95% CI [0.345, 0.749]; symptoms of aggressiveness β = 0.394, *p* < .001, 95% CI [0.697, 1.287]). The results furthermore suggested that parental mental health symptoms mediate the association between psychosocial work stress and parent-child bonding (symptoms of depression, ab = 2.491, 95% CI [1.472, 3.577] and of aggressiveness, ab = 2.091, 95% CI [1.147, 3.279]). The mediation effect was also found in the separate analyses for the mothers and fathers.

**Discussion:**

The results of this study during the early COVID-19 pandemic in Germany highlight the importance of prevention as well as intervention measures in relation to psychosocial work stress that may play a debilitating role in the context of family relationships. In addition, the results suggest that both employers and employees should be made aware of the importance of psychosocial work stress, as it can have a negative impact on mental health, which in turn may have a major influence on family relationships.

## Introduction

Parent-child bonding has been suggested to be the “central and most important psychological process of the puerperium” [1] and to have a major impact on the young child’s development [2, 3]. Bonding refers to the relationship with the child from a parent’s perspective and can be “described as the quality of the emotional tie from the parent to the child” [2]. The term is used synonymously with parent-child relationship both in the literature [4] as well as in this paper.

A sensitive and responsive family environment fosters the development of secure parent-child bonding [5, 6]. Maternal and paternal sensitivity to their infant’s needs, immediate responsiveness to distress, and interactional synchrony are supportive regarding this process [7]. This, in turn, contributes to a child’s healthy development during the first years of life in terms of behavior, emotions, and mental health [2, 8, 9].

On the other hand, work plays a major role in life and can have a significant, beneficial impact on health and well-being. It provides regular income, social inclusion, and a chance for personal growth [10]. However, work can also be a cause for stress. The concept of psychosocial work stress can be shown by the effort-reward-imbalance (ERI) model [10]. According to the ERI model, effort at work implies demands such as workload, interruptions, and time pressure. Rewards, in turn, are provided in forms of salary, esteem, career opportunities, and job security. An imbalance between (high) efforts and (low) rewards may result in a higher risk of adverse mental health outcomes [11, 12].

Stress can spill over from one area of life to another area in relation to a specific individual, such as in the transmission of stress from one domain (work) to another one (family) [13]. It is presumed that high work demands prevent parents from spending a lot of time with their offspring, causing family relationships to suffer [14]. Studies have shown that job stress, job demands, and work characteristics (such as an organization that is not family-friendly) are associated with work-family conflict, defined as the incompatibility between work and family [15, 16].

Regarding the parent-child relationship, it was found that work stress has a negative effect on parenting behavior [17]. Factors such as prolonged hours at the workplace and work overload have shown an association with a low-quality parent-child relationship [18]. A recent study examining spillover effects found that job insecurity is negatively related to parent-child bonding [7]. It has been shown that for both mothers and fathers, behavioral and emotional withdrawal occur on days with high demand or interpersonal stress at work, when they appear to be less emotionally involved with their children [19, 20]. However, as of yet, there are no studies which investigate psychosocial working conditions modelled by ERI and parent-child bonding.

A spillover effect from work to the family domain occurred during the COVID-19 pandemic but the effect of the pandemic on the work-family interface is still unknown [21]. In the wake of the global spread of the SARS-CoV-2 and subsequent containment efforts, workers had to cope with numerous consequences in the work domain. There was an increase in job insecurity and job losses, and the need to work from home [22, 23]. In the family domain, mothers and fathers were exposed to increased stress and responsibilities at home [24, 25]. The situation was particularly precarious for parents who continued to work full time but were unable to take advantage of emergency care for their children. Boundaries between the work and home domain tended to diminish, potentially fueling work-family conflict. Taken together, the parents’ perception of the COVID-19 pandemic as a stressor may be associated with increased parenting stress and thus in turn, with an increased risk of harsh parenting and an impaired parent-child relationship or even child abuse [26, 27].

Not only parental work stress, but also the mother’s and father’s mental health constitutes a possible factor which may compromise parent-child bonding [28]. This would also represent a crossover effect in the sense of an inter-individual transmission of stress (parent-child) within one domain (family). In this case, stress spills over from one person (mother/father) to another person (child), within one area of life (family).

Parents’ mental health could be particularly strained in the wake of pandemic conditions. Compared to the time before the pandemic, mental health declined after the outbreak of the pandemic in 2020 [29]. Studies have also suggested that impaired mother-child bonding is related to maternal depression [30, 31]. Mothers with depressive symptoms are more likely to exhibit distant behaviors towards their child (e.g., less affectionate touch [32], fewer vocal and visual interactions [33]) than mothers without depressive symptoms. Since affectionate mother-child interaction goes along with strong mother-child bonding, the latter may be diminished in mothers suffering from depressiveness [34]. A German study supported these results and highlighted the importance of the mother’s mental state in the first year of the child’s life [35]. A Swedish study extended the research to both parents and found that not only the mother’s but also the father’s depressiveness is related to dysfunctional bonding [36].

Besides affective disorders, it is necessary to address the issue of aggression in the family domain in order to protect everyone involved. Distress reactions like anger are likely to occur during the exceptional time of the COVID-19 pandemic [37]. Several studies have indicated that parent-child relationships in families suffering from a violent or aggressive family member are severely disrupted [38, 39]. In the case where the father is the abusive parent, not only the relationship between the violent father and his children is disturbed. His aggressiveness may also have negative consequences for the mother-child bonding, as she is often traumatized by the partner’s abusive behavior and therefore limited in her ability to emotionally care for the children [40].

Research on mother-child violence and its impact on the mother-child bonding is scarce and tends to focus on child outcomes like internalizing or externalizing behavior or delayed cognitive development [41, 42]. A possible explanation for the scarcity may be that motherly violence against their offspring is more of a taboo, since mothers are considered as less aggressive and as those who take the protective and caring role in the family [43]. Furthermore, studies have suggested that it is often the father who is the abusing parent [44].

### Summary of objectives

**Fig. 1:**
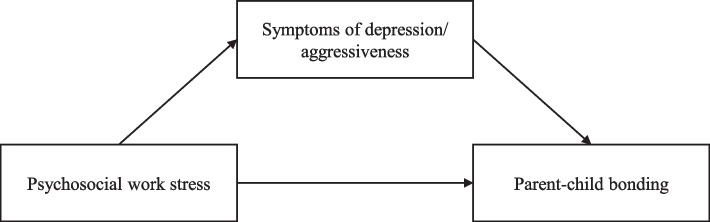
The hypothesized associations between psychosocial work stress, symptoms of depression/aggressiveness, and parent-child bonding

The possible links between the work and the family domain in an unprecedented context like the COVID-19 pandemic need further investigation. This study aims to examine the association between psychosocial work stress and parent-child bonding in a community sample of mothers and fathers. It is hypothesized that higher levels of work stress are associated with weaker parent-child bonding (hypothesis 1) and that higher levels of work stress are associated with a higher score of symptoms of depression and aggressiveness (hypothesis 2). Moreover, it is hypothesized that a higher score of symptoms of depression and of aggressiveness is associated with weaker parent-child bonding (hypothesis 3). Finally, the study also hypothesizes that the association between psychosocial work stress and parent-child bonding is mediated by symptoms of depression and aggressiveness (hypothesis 4; see Fig. 1). In addition, this study examines in an exploratory manner differences between mothers and fathers: are there any differences in terms of potential associations between psychosocial work stress, symptoms of depression/aggressiveness, and parent-child bonding, and if so, to what extent? The mediation analyses are explored in this regard as well: are there any differences between mothers and fathers, and if so, to what extent?

## Methods

### Design

The prospective cohort study “Dresden Study on Parenting, Work, and Mental Health” (**DR**esdner Studie zu **E**lternschaft, **A**rbeit und **M**entaler Gesundheit, **DREAM**) examines the relationship between parental work participation, role distribution, stress factors, and their effects on family mental and somatic health [45]. Recruitment in the main study, using the convenience sampling method, lasted from June 2017 until end of 2020. The community sample of this study consists of women who were pregnant at the time of recruitment and their partners, at that time living in Dresden, Germany, or in the surrounding area. For the DREAM study, the participants completed questionnaires on various physical and mental health outcomes with the choice of filling them out on paper or online. The DREAM study has currently six measurement points: T1 during pregnancy, T2 at 8 weeks, T3 at 14 months, T4 at 2 years, T5 at 3 years, and T6 at 4.5 years after birth. Further information on the DREAM study is provided in the corresponding study protocol [45].

As an addition to the regular measurement points, a subsample of the main DREAM study was invited to take part in the longitudinal online sub-study DREAM_CORONA_. The DREAM_CORONA_ sub-study investigates experiences of (expectant) parents during the COVID-19 pandemic (e.g., isolation, school and daycare closures, working in home office) and its impact on family health, role distributions, and relationships. Pandemic restrictions hindered sending out study material and thereby reaching participants using the paper-pencil version. Due to feasibility reasons, parents of twins or multiples did not receive an invitation. Thus, only online participants with a singleton pregnancy got an invitation. Dream_CORONA_ has two measurement points, and the present paper reports results of the first measurement point of this sub-study. 1885 persons were invited to participate between May 12 to October 1, 2020. From those, 1057 took part in Dream_CORONA_, resulting in a response rate of 56.1%. All data were derived from the DREAM_CORONA_ sub-study except for the information about parents' education, which was taken from the main DREAM study. Data were stored on the Research Electronic Data Capture (REDCap) data management platform, which is a web-based software for secure data collection and organization [46]. REDCap is hosted at the “Koordinierungszentrum für Klinische Studien” at the Faculty of Medicine of the Technische Universität Dresden.

### Sample

In the current study, female partners of mothers were excluded, in order to prevent interference between the group of mothers and their partners, when the results of the data analyses were stratified by sex to uncover possible group differences. Inclusion criteria comprised provision of informed consent and completing the questionnaire until 5th of June, 2020, because afterwards new COVID-19 regulations came into effect in the concerned region. Main COVID-19 policies from 20th of April up to that point affected workplaces as well as childcare and education institutions in Germany. For instance, except frontline workers, employees were required to work in home office unless there was at least 10 square meters of space per person [47]. After closure during the first lockdown in Germany for the vast majority of the population (10th of March until 19th of April, 2020), childcare institutions and schools gradually opened again (graduation classes of elementary schools at the beginning of May); all classes of elementary schools and daycare in the middle of May [48]. However, hygiene rules such as regular washing of hands had to be followed during school time and special events. For instance, sport events were still not carried out.

**Fig. 2:**
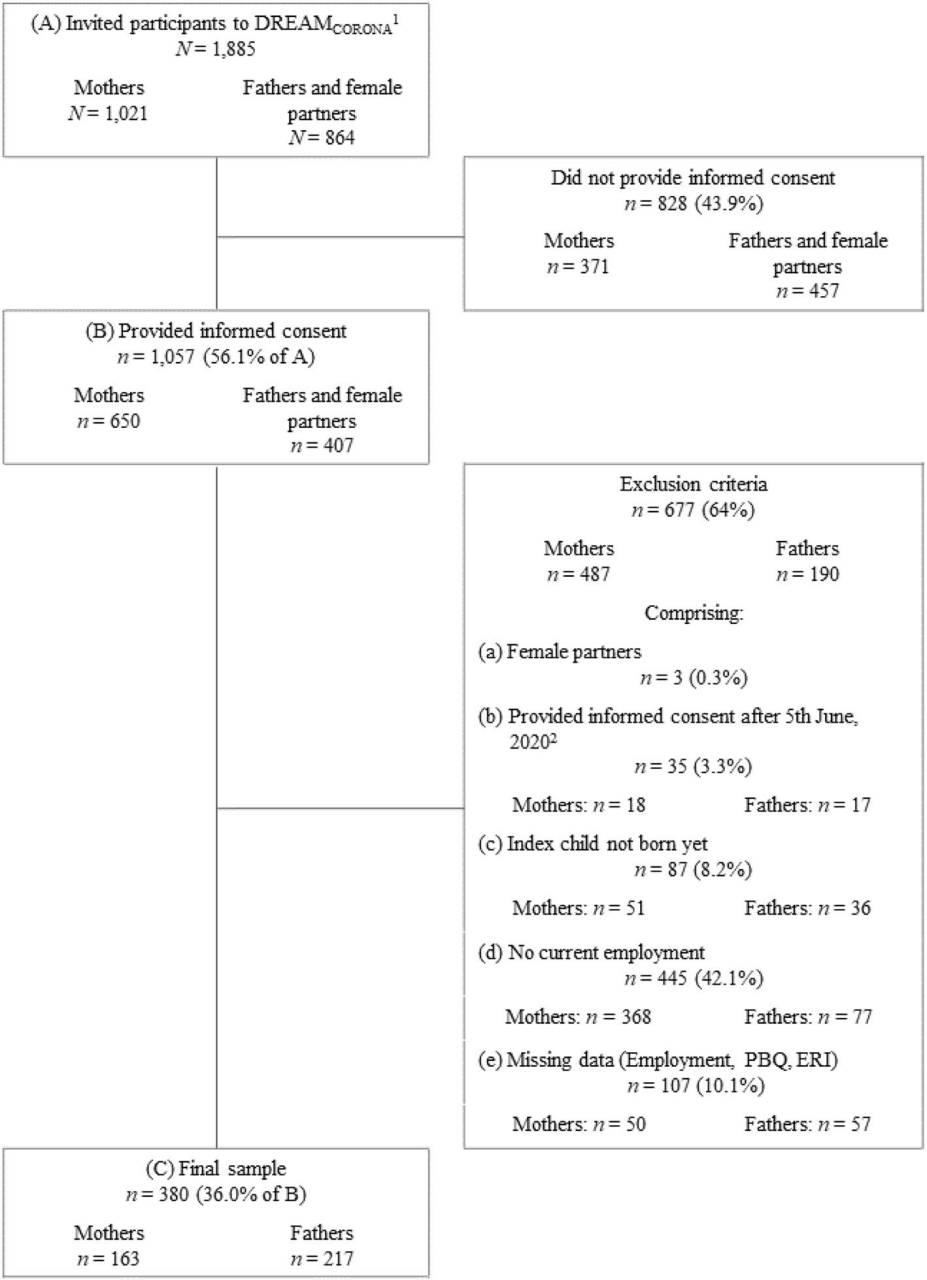
Flowchart of retention rate and exclusion criteria resulting in final sample. *Note*. ^1^Online participants of the general DREAM study as of April 2020 (twin and multiple pregnancies excluded). ^2^With 6th June, 2020 new COVID-19 regulations came into effect. PBQ = Postpartum Bonding Questionnaire. ERI = Effort-Reward Imbalance Questionnaire

Participants had to be currently employed (i.e., working full-time, part-time, irregularly, or being marginally employed, undergoing an apprenticeship or federal voluntary work) in order to evaluate psychosocial work stress. Parents who provided illogical information regarding their employment situation (working zero hours per week or those who reported simultaneously being on parental leave and being employed full-time) were excluded. Participants were asked to specifically refer to their current working conditions since February 2020, i.e., the time when the first effects of the impending pandemic emerged in this region. Moreover, the index child (i.e., the child with whom the parents first took part in the study) had to be born before 20th of April, 2021 in order to measure parent-child bonding as the outcome variable. The final sample consisted of 380 participants (163 mothers and 217 fathers). The flowchart with the retention rate and exclusion criteria resulting in the final sample is shown in Fig. 2.

### Measures

#### Measure for the predictor variable psychosocial work stress: Effort-Reward Imbalance Questionnaire

Psychosocial work stress was assessed with the Effort-Reward Imbalance Questionnaire (ERI), which is a self-rating scale [49]. It measures chronic work-related stress as an imbalance between high efforts spent for the job – for instance, high work performance and low rewards like poor opportunities for advancement. It consists of three sub-scales: effort, reward, and overcommitment. In this study, the German short version with the effort and reward subscale was used [49]. It contains ten items on a Likert scale from 1 (*strongly agree*) to 4 (*strongly disagree*) out of which four items are inverted. The range of the effort scale with three items is 3 to 12 (item example “I have constant pressure due to a heavy workload.”), and of the reward scale with seven items 7 to 28 (item example “Considering all my efforts and achievements, my salary/income is adequate.”). The sum scores of these ratings were recoded for the analyses, so that high scores on each scale reflect high effort and reward. The overall quotient was calculated by dividing effort by reward, then multiplied by the ratio of the number of items – three out of seven [49]. The higher the imbalance between (high) expenditure and (low) reward, the higher the measure of work stress.

Missing item values on the reward scale were replaced by the mean value if not more than three items were missing. All three items of the effort scale had to be answered in order for ERI to be evaluated. In this sample, the reliability of the effort scale was questionable (Cronbach’s α = .66), and that of the reward scale was acceptable (Cronbach’s α = .78).

#### Measure for the outcome variable parent-child bonding: Postpartum Bonding Questionnaire

Parent-child bonding was measured by the Postpartum Bonding Questionnaire (PBQ), which is a self-rating screening instrument for bonding disorders [4]. In this study, the German version was used and completed by both, mothers and fathers. The questionnaire consists of 25 items on a Likert scale from 0 (*always*) to 5 (*never*) out of which eight items are inverted [4]. It contains four sub-scales, for each of which a sub-score can be calculated: impaired bonding (twelve items; example “I feel close to my baby.”), rejection and anger (seven items; example “My baby irritates me.”), anxiety about care (four items; example “I am afraid of my baby.”), risk of abuse (two items, score range 0–10; example “I feel like hurting my baby.” [4]. In the current study, the sum score (0–125) was used with a higher value indicating weaker parent-child bonding. The reliability of the PBQ in this sample was good (Cronbach’s α = .83).

#### Measures for potential mediator variables

Symptoms of depression were measured by the German version of the Edinburgh Postnatal Depression Scale (EPDS) [50]. This self-rating questionnaire records symptoms of depression in the past week. Each of the ten items, seven of which are reverse scored, offers four response options on a Likert scale from 0 to 3 (item example “I was sad and miserable.”). The higher the sum score, the more severe the symptoms of depression. The reliability of the EPDS in the present sample was good (Cronbach’s α = .84).

Symptoms of aggressiveness were measured by the sub-scale anger-hostility of the Symptom-Check-List-90-R (SCL-90-R) [51]. The SCL-90-R is a self-assessment questionnaire for detecting mental distress regarding the past week and consists of nine separate scales and 90 items. The answers are given on a Likert scale from 0 (*not at all*) to 4 (*very strongly*). A higher sum score indicates a higher level of mental distress. The sub-scale anger-hostility used in this study has a total of six items (example “How much did you suffer from feeling easily irritated or upset?”) and measures anger-hostility defined as “irritability and imbalance up to strong aggressiveness with hostile aspects” [51]. Its reliability in the study sample was good (Cronbach’s α = .81).

Missing items in the PBQ, EPDS, and the anger-hostility sub-scale of the SCL-90-R were replaced by the mean value of the respective participant if a maximum of 20% of the items were not completed.

#### Measures for potential confounders

Several variables were considered as potential confounders in our analyses, as they may be associated with work stress and/or parent-child bonding: Education, number of children, age of index child, hours of childcare, hours of household work, hours of work, and working in home office. They were selected based on previous research and correlation analyses with the predictor and outcome variables. Education was used as a measure for the socioeconomic status. It was assessed during pregnancy with the index child using a question regarding professional qualification in the main DREAM study questionnaire: “Do you have a university degree: yes or no?”. The other variables were collected as items in the DREAM_CORONA_ questionnaire. The participants were asked to fill out the age of the index child and the total number of their children. Concerning work factors, participants were asked to provide information about the number of hours of work (per week), number of hours of childcare and of household work (per workday), and to indicate if they work in home office due to the COVID-19 restrictions. The variable parents’ sex was not included as a confounder, because the results of the data analyses were investigated separately for mothers and fathers.

### Statistical analyses

All statistical analyses were conducted using the software IBM SPSS Statistics (Version 27). Descriptive analyses were carried out for demographic characteristics of the sample (sex, age, employment status), for the potential confounders, as well as for the predictor and the outcome variables. Pearson and Kendall-Tau-b correlation analyses were performed in order to detect statistically significant confounders for the regression model.

Linear regression analyses were calculated to investigate possible associations between psychosocial work stress, psychological health factors (symptoms of depression and aggressiveness), and parent-child bonding. Standardized regression coefficients were calculated. The regression analyses were computed with and without potential confounders to check for possible differences. Finally, mediation analyses were performed in order to test the mediation effect of psychological health factors (symptoms of depression and aggressiveness) between psychosocial work stress and parent-child bonding. The mediation analyses were carried out once without and once with the potential confounders to investigate possible differences.

The linear regression as well as the mediation analyses were conducted with the SPSS modeling tool PROCESS v.3.5, which uses ordinary least squares regression to estimate model coefficients, standard errors, *p*-values, and confidence intervals [52]. For the present study, bootstrapping with 5000 iterations was used. Heteroscedasticity consistent standard errors [53] and 95% percentile confidence intervals were calculated. Due to missing data in several variables, *n* varied slightly between the different analyses.

## Results

### Sample description

The final sample consisted of 380 parents (163 mothers, 217 fathers). The characteristics of the sample are provided in Table 1. The mean age of the parents was 33.88 years (*SD =* 4.59; *Range* = 24–55). Almost half of the participants (46.8%) had a university degree. The mean number of children in a family was 1.23 (*SD* = 0.51; *Range* = 1–4) and the age of the index child averaged 21.5 months. On average, mothers worked 29 hours, whereas fathers worked 35 hours per week. More than half of the parents (63.2%) reported to work in home office due to the COVID-19 restrictions.

**Table 1:** Sample characteristics

	Mothers	Fathers	Total sample
	*n *= 163	*n *= 217	*n *= 380
Psychosocial work stress	0.95 ± 0.38 (0.26–2.00)	0.94 ± 0.36 (0.28–3.67)	0.94 ± 3.37 (0.26–3.67)
Symptoms of depression	6.93 ± 4.68 (0–22)	4.68 ± 4.23 (0–20)	5.65 ± 4.56 (0–22)
Symptoms of aggressiveness	3.50 ± 3.46 (0–19)	1.95 ± 2.56 (0–15)	2.60 ± 3.07 (0–19)
Parent-child bonding	13.95 ± 8.74 (0–41)	12.03 ± 7.54 (0–43)	12.85 ± 8.12 (0–43)
Age	32.98 ± 3.87 (25–43)	34.55 ± 4.96 (24–55)	33.88 ± 4.59 (24–55)
Education			
No university degree	82 (50.3)	120 (55.3)	202 (53.2)
University degree	81 (49.7)	97 (44.7)	178 (46.8)
Age of index child	21.50 ± 6.55 (1–34)	14.63 ± 9.06 (0–32)	17.58 ± 8.74 (0–34)
0–12 months	16 (9.8)	101 (46.5)	117 (30.8)
13–24 months	88 (54.0)	75 (34.6)	163 (42.9)
25–36 months	59 (36.2)	41 (18.9)	100 (26.3)
Number of children	1.22 ± 0.46 (1–4)	1.24 ± 0.56 (1–4)	1.23 ± 0.51 (1–4)
1	129 (79.1)	174 (80.2)	303 (79.7)
2	33 (20.2)	35 (16.1)	68 (17.9)
3	0 (0.0)	6 (2.8)	6 (1.6)
4	1 (0.6)	2 (0.9)	3 (0.8)
Hours of work^a^	28.58 ± 10.77 (2–55)	35.0 ± 12.17 (2–72)	32.29 ± 12.02 (2–72)
Childcare^b^	4.92 ± 2.19 (1.5–22)	2.85 ± 1.58 (0–9)	3.72 ± 2.12 (0–22)
Household work^b^	2.04 ± 1.38 (0–13)	1.70 ± 1.30 (0–8.5)	1.85 ± 1.34 (0–13)
Home office			
No	61 (37.4)	79 (36.4)	140 (36.8)
Yes	102 (62.6)	138 (63.6)	240 (63.2)

*Note*. *n* (%) or *M* ± *SD* (*Range*). Psychosocial work stress (quotient of ERI = Effort-Reward Imbalance Questionnaire), Symptoms of depression (sum score of EPDS = Edinburgh Postnatal Depression Scale), Symptoms of aggressiveness (sum score of SCL-90-R = Symptom-Check-List-90-Revised, sub-scale anger-hostility), Parent-child bonding (sum score of PBQ = Postpartum Bonding Questionnaire). ^a^Hours per week, ^b^Hours per day

### Correlation analyses

Correlation analyses between the predictor, mediator, outcome, and potential confounders were carried out in order to investigate possible associations (Table 2). Based on these analyses, the selected confounders comprised age of index child (in months), hours of work (per week), childcare (at home per day), and work in home office (yes/no).

**Table 2:** Correlation matrix including the predictor, mediator, outcome variables, and potential confounders of the total sample

	1.	2.	3.	4.	5.	6.	7.	8.	9.	10.	11.
1. Psychosocial work stress	–										
2. Symptoms of depression	.339^**^	–									
3. Symptoms of aggressiveness	.225^**^	.604^**^	–								
4. Parent-child bonding	.128^*^	.362^**^	.420^**^	–							
5. Education (university degree)	−.003	.032	−.011	.017	–						
6. Age of index child	.027	.078	.106^*^	.148^**^	.017	–					
7. Number of children	.062	.064	.097	.010	.017	.042	–				
8. Hours of work	.075	−.171^**^	−.181^**^	−.154^**^	−.048	−.099	−.004	–			
9. Childcare	−.051	.170^**^	.295^**^	.123*	.033	.241^**^	−.007	−.360^**^	–		
10. Household work	.045	.153^**^	.204^**^	.017	−.088	.059	.047	−.104^*^	.197^**^	–	
11. Home office (yes)	.007	.186^*^	.028	.152^**^	.184^**^	.048	−.075	−.106^*^	.044	.061	–

Kendall-Tau-b correlation coefficients were computed for the potential confounders education and home office. Pearson correlation coefficients were computed for all other variables. Statistically significant correlations of potential confounders with the outcome variable PBQ are printed in bold. Psychosocial work stress (quotient of ERI = Effort-Reward Imbalance Questionnaire), Parent-child bonding (sum score of PBQ = Postpartum Bonding Questionnaire), Symptoms of depression (sum score of EPDS = Edinburgh Postnatal Depression Scale), Symptoms of aggressiveness (sum score of SCL-90-R = Symptom-Check-List-90-Revised, sub-scale anger-hostility). ^*^p < .05. ^**^p < .01. ^***^p < .001

### Regression and mediation analyses

#### Analyses with the mediator variable symptoms of depression

**Fig. 3:**
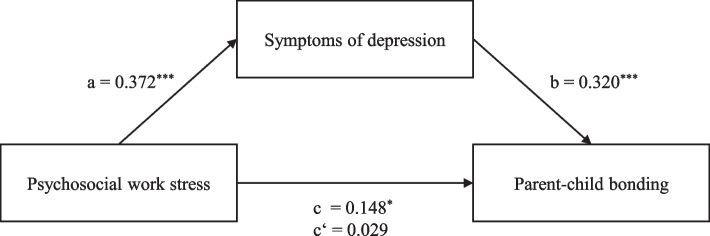
Standardized regression coefficients for the associations between psychosocial work stress, symptoms of depression, and parent-child bonding for the total sample (controlled for potential confounders). Note. c = total effect; c’ = direct effect. ^*^ = *p* < .05. ^**^ = *p* < .01. ^***^ = *p* < .001

In the model with symptoms of depression as the mediator (Fig. 3), the total effect of psychosocial work stress on parent-child bonding was not statistically significant in the unadjusted analysis (β = 0.128, *p* = .053, 95% CI [−0.029, 5.369]; Model 1, Table 3). However, after adding the potential confounders to the model, the association between psychosocial work stress and parent-child bonding was statistically significant (β = 0.148, *p* = .017, 95% CI [0.566, 5.614], Model 2, Table 3; hypothesis 1). This means, the total effect of psychosocial work stress on parent-child bonding was statistically significant. Additionally, an older age of the index child and working in home office (both added as confounders) significantly predicted parent-child bonding in the multiple regression model.

**Table 3:** Predictive value of psychosocial work stress on parent-child bonding for the total sample

Variable	*B*	*SE B*	β	95% CI	*p*
**Model 1 (*R***^***2***^ **= 0.02)**					
Constant	10.051	1.299		[7.497, 12.605]	< .001
Psychosocial work stress	2.670	1.373	0.128	[-0.029, 5.369]	.053
**Model 2 (*R***^***2***^ **= 0.08)**					
Constant	8.194	2.406		[3.463, 12.925]	.001
Psychosocial work stress	3.090	1.284	**0.148**	[0.566, 5.614]	**.017**
Age of index child^a^	0.109	0.051	**0.123**	[0.008, 0.209]	**.035**
Hours of work^b^	-0.073	0.039	-0.114	[-0.150, 0.003]	.059
Childcare^c^	0.184	0.350	0.051	[-0.503, 0.872]	.599
Home office (yes)	2.028	0.780	**0.127**	[0.494, 3.562]	**.010**

*Note*. *B* = unstandardized regression coefficient; *SE B* = standard error, based on 5,000 bootstrap samples; β = standardized beta coefficient, CI = confidence interval with α = 0.05, 95% percentile, based on 5,000 bootstrap samples; Statistically significant associations (*p* < .05) are marked in bold; Psychosocial work stress (quotient of ERI = Effort-Reward Imbalance Questionnaire); Home office coded as 0 = not working from home, 1 = working from home. ^a^Age in months, ^b^Hours per week, ^c^Hours per day

Higher levels of psychosocial work stress also significantly predicted higher scores of symptoms of depression (β = 0.372, *p* < .001; hypothesis 2). These in turn significantly predicted weaker parent-child bonding (β = 0.320, *p* < .001; hypothesis 3). Moreover, tests of indirect effects indicated that the association between psychosocial work stress and parent-child bonding was mediated by symptoms of depression (indirect effect ab = 2.491, 95% CI [1.472, 3.577]; hypothesis 4). Additionally, after including symptoms of depression as a mediator, the relationship between psychosocial work stress and parent-child bonding was no longer statistically significant (β = 0.029, *p* = .665; data not shown).

#### Analyses with the mediator variable symptoms of aggressiveness

**Fig. 4:**
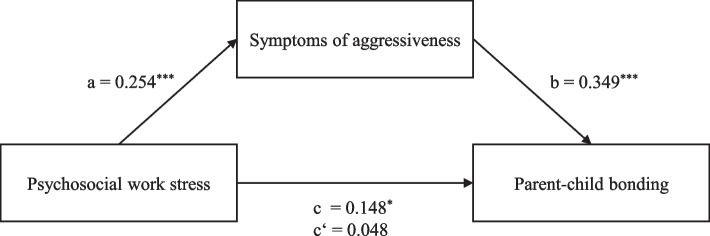
Standardized regression coefficients for the associations between psychosocial work stress, symptoms of aggressiveness, and parent-child bonding for the total sample (controlled for potential confounders). Note. c = total effect; c’ = direct effect. ^*^ = *p* < .05. ^**^ = *p* < .01. ^***^ = *p* < .001

In the model with symptoms of aggressiveness as the mediator (Fig. 4), the total effect of psychosocial work stress on parent-child bonding was statistically significant (β = 0.148, *p* = .017; Model 2, Table 3; hypothesis 1). Higher levels of psychosocial work stress also significantly predicted higher scores of symptoms of aggressiveness (β = .254, *p* < .001; hypothesis 2). These in turn significantly predicted weaker parent-child bonding (β = 0.394, *p* < .001; hypothesis 3). Moreover, tests of indirect effects indicated that the association between psychosocial work stress and parent-child bonding was mediated by symptoms of aggressiveness (indirect effect ab = 2.091, 95% CI [1.147, 3.279]; hypothesis 4). Furthermore, after including symptoms of aggressiveness as a mediator, the relationship between psychosocial work stress and parent-child bonding was no longer statistically significant (β = 0.048, *p* = .369).

#### Regression and mediation analyses stratified for parents’ sex

The analyses were then evaluated separately for mothers and fathers in order to explore differences between the sexes. The same potential confounders were considered in the analyses.

There was no association between psychosocial work stress and parent-child bonding in the models calculated for the mothers (unadjusted model, β = 0.130, *p* = .104, 95% CI [−0.585, 6.219], adjusted model: β = 0.160, *p* = .060, 95% CI [−0.129, 7.070], Table 4). For the group of fathers, the association between psychosocial work stress and parent-child bonding was not statistically significant for both models as well (unadjusted: β = 0.124, *p* = .260, 95% CI [−1.839, 6.772]; adjusted: β = 0.132, *p* = .195, 95% CI [−1.354, 6.584]; Table 4).

**Table 4:** Predictive value of psychosocial work stress on parent-child bonding. Results stratified for parents’ sex. Standardized regression coefficients for the associations between psychosocial work stress, symptoms of aggressiveness, and parent-child bonding for the total sample (controlled for potential confounders)

	Mothers					Fathers				
Variables	*B*	*SE B*	β	95% CI	*p*	*B*	*SE B*	β	95% CI	*p*
Model 1 (*R*^*2*^ = 0.02/0.02^a^)										
Constant	10.941	1.684		[7.616, 14.267]	< .001	9.471	2.021		[5.487, 13.454]	< .001
Psychosocial work stress	2.817	1.722	0.130	[−0.585, 6.219]	.104	2.467	2.184	0.124	[−1.839, 6.772]	.260
Model 2 (*R*^*2*^ = 0.14/0.04^a^)										
Constant	9.945	5.087		[−0.106, 19.996]	.052	7.187	3.100		[1.077, 13.298]	.021
Psychosocial work stress	3.470	1.822	0.160	[−0.129, 7.070]	.060	2.615	2.013	0.132	[−1.354, 6.584]	.195
Age of index child^b^	0.178	0.112	0.136	[−0.044, 0.400]	.115	0.087	0.055	0.111	[−0.021, 0.194]	.115
Hours of work^c^	−0.199	0.071	**−0.252**	[−0. 340, −0.059]	**.006**	−0.011	0.045	−0.019	[−0.099, 0.077]	.802
Childcare^d^	0.079	0.767	0.021	[−1.437, 1.594]	.918	0.153	0.434	0.034	[−0.703, 1.008]	.725
Home office (yes)	3.104	1.297	**0.181**	[0.536, 5.672]	**.018**	1.358	1.000	0.092	[−0.614, 3.331]	.176

*B* = unstandardized regression coefficient; *SE B* = standard error, based on 5000 bootstrap samples; β = standardized beta coefficient; CI = confidence interval with α = 0.05, 95% percentile, based on 5000 bootstrap samples; statistically significant associations (*p* < .05) are marked in bold; Psychosocial work stress (quotient of ERI = Effort-Reward Imbalance Questionnaire); Home office coded as 0 = not working from home, 1 = working from home

^a^*R*^*2*^ = mothers/fathers

^b^Age in months

^c^Hours per week

^d^Hours per day

**Fig. 5:**
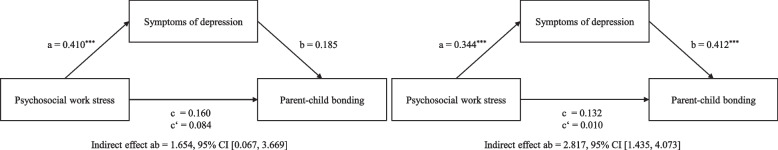
Standardized regression coefficients for the associations between psychosocial work stress, symptoms of depression, and parent-child bonding (controlled for potential confounders) for the group of mothers and fathers. Note. c = total effect; c’ = direct effect. ^*^ = *p* < .05. ^**^ = *p* < .01. ^***^ = *p* < .001

In the first mediation model stratified for the parents’ sex (see Fig. 5) higher levels of psychosocial work stress significantly predicted higher scores of symptoms of depression for the mothers as well as the fathers. They in turn significantly predicted a higher score of (i.e., weaker) parent-child bonding for the fathers, but not for the mothers. There was a mediation effect of symptoms of depression for both parents. Furthermore, after including symptoms of depression as a mediator, the relationship between psychosocial work stress and parent-child bonding remained statistically not significant for both parents.

**Fig. 6:**
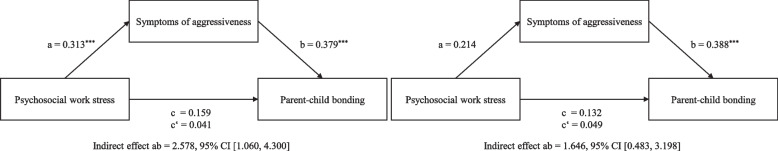
Standardized regression coefficients for the associations between psychosocial work stress, symptoms of aggressiveness, and parent-child bonding (controlled for potential confounders) for the group of mothers and fathers. Note. c = total effect; c’ = direct effect. ^*^ = *p* < .05. ^**^ = *p* < .01. ^***^ = *p* < .001

In the second mediation model stratified for the parents’ sex (see Fig. 6) higher levels of psychosocial work stress significantly predicted higher scores of symptoms of aggressiveness for the mothers, but not for the fathers. They in turn significantly predicted a higher score of (weaker) parent-child bonding and there was a mediation effect of symptoms of aggressiveness for both parents. Furthermore, after including symptoms of aggressiveness as a mediator, the relationship between psychosocial work stress and parent-child bonding remained statistically not significant for both parents.

## Discussion

The present study aimed to investigate a possible association between parental psychosocial work stress and parent-child bonding during the early COVID-19 pandemic in Germany. In the total sample, an association was only found after adjusting for education, number of children, age of index child, hours of childcare, hours of household work, hours of work, and working in home office, indicating that higher psychosocial work stress is associated with weaker bonding between the parent and child. The separate analyses for mothers and fathers did not reveal a statistically significant relationship between psychosocial work stress and parent-child bonding. In the total sample, the higher the psychosocial work stress was, the higher were the parental symptoms of depression and aggression. These in turn were related to weaker parent-child bonding. The results furthermore suggested that parental mental health symptoms of depression and aggressiveness mediate the association between psychosocial work stress and parent-child bonding. The mediation effect was found in the overall sample and, moreover, in the separate analyses for mothers and fathers.

### Association between psychosocial work stress and parent-child bonding

Parental psychosocial work stress and parent-child bonding showed a statistically significant association after adjusting for confounders, indicating a weakened parent-child bonding with increasing psychosocial work stress (along with hypothesis 1). Since no studies have been conducted to explicitly examine the relationship between psychosocial work stress and parent-child bonding, the results of the present study expand this area of research.

The separate analyses for mothers and fathers did not reveal a statistically significant association between psychosocial work stress and parent-child bonding. This may be due to a lack of power, as the sample size was roughly halved in the separate analyses for mothers and fathers.

### Association between psychosocial work stress and symptoms of impaired mental health

Psychosocial work stress predicted higher scores of symptoms of depression as well as of aggressiveness (along with hypothesis 2), which aligns very well with prior research [54, 55]. This result was true for the whole sample as well as for the analyses conducted separately for mothers and fathers (the only exception was that psychosocial work stress narrowly failed to significantly predict paternal symptoms of aggressiveness). Work-related stressors can contribute to an increasingly negative attitude toward work. High job demands and possible additional stress during the COVID-19 pandemic, without adequate employer rewards, may affect mental health. This can be accompanied by exhaustion and dejection, or irritability and imbalance. These factors possibly promote adverse mental health, including symptoms of depressiveness and aggressiveness [56].

### Association between symptoms of impaired mental health and parent-child bonding

Symptoms of depression as well as aggressiveness predicted weaker parent-child bonding in the analyses for the total sample (hypothesis 3 is met), which ties in with previous research results [29, 36, 40]. Russell and colleagues collected data during the first few months of the COVID-19 pandemic and found that there is a clear relationship between mental health symptoms and both parent-child conflict and closeness [26]. Parents with more severe symptoms of depression reported greater conflict with their children, which assumingly has a straining impact on family relationships. Such effects on the family can also be expected from aggressive behavior. If parents show angry or violent behavior towards their child, this disturbs trust, and the bond may suffer accordingly.

The separate analyses for mothers and fathers showed differences in their results. Maternal symptoms of depression unexpectedly did not predict bonding with their child, which is contrary to former research that has shown that mothers with depression experienced weaker bonding with their child [28, 30, 35]. For instance, mothers with higher depressive symptomatology reported more conflict in their relationship to their child [26]. For fathers, on the other hand, the picture presented itself to be quite different in the present study. For them, symptoms of depression were associated with bonding with the child. Russell and colleagues also found that depressive fathers reported a higher score of conflict with their children than depressive mothers [26]. Mental health and parent-child bonding should be addressed in future research and investigated in more depth, especially concerning differences between mothers and fathers.

### The mediator role of parental impaired mental health

There was a mediation effect for symptoms of depression as well as aggressiveness in the analyses of the total sample (along with hypothesis 4) as well as in the separate analyses for mothers and fathers. Hence, the results of this study highlight obviously not only a spillover effect from the work to the family domain – namely, that work stress spills over to the family impacting the relationships of parents and their children. In addition, there also appears to be a crossover effect within the family mediated by the mental health status of the parents. Work stress can cross from mentally distressed parents to their children and affect the parent-child bonding [13].

The findings regarding the mediating role of parental depressiveness fit with previous research. For instance, studies have shown that on days with higher loads of work, depressive mothers state less involvement with and less responsiveness to their children [19] and that parental depressive symptoms mediate the association between work stress and parent-adolescent relationships [57]. Research on the work-family interface that examines a possible mediating role of parental aggressiveness for the association between work stress and parent-child bonding is very scarce. It tends to focus on a possible mediating role of poor parenting behavior like using authoritarian disciplinary strategies [58]. The results of the present study therefore highlight the importance of addressing parental emotional dysregulation and aggressive behavior in future research.

One explanation for the mediating role of mental health symptoms in the association between parental work stress and the relationship with children may lie in the cognitive and emotional distress that is often part of depressive and aggressive symptomatology. If parents experience stress at work and become depressed or emotionally agitated as a result, they tend to be mentally and emotionally preoccupied with the sources of stress. Attention to the well-being of other family members, especially children, may suffer as mother and father are consumed with their own problems. Parents, however, who are sensitive and attentive to their children in everyday life can lay a good foundation for close parent-child bonding [59]. In this context, Moreira and her colleagues described the value of mindful parenting, which is characterized by responsiveness, attentiveness, openness towards and acceptance of the child [60]. Furthermore, mindful parents are able to regulate emotions and behavior while interacting with their child. This may be exactly what is challenging for parents who are under psychosocial work stress in the extremely demanding context of the COVID-19 pandemic, and struggle with depressive or aggressive symptomatology and thus, strengthening a bond with their child may be disrupted [60].

### Strengths and limitations

This study extends the research on the work-family interface. The present work was, to the best of our knowledge, the first to address the relationship between psychosocial work stress and parent-child bonding. Secondly, this study was among the first to investigate this particular question in the wake of the COVID-19 pandemic. The aspect of mental health was included and looked at in more detail, as this was found to be important in previous studies in contexts of crisis. Thirdly, this study adds to international research concerning work stress and its impact on families, because so far there are only a few other recent studies that explicitly addressed parents in Germany [61, 62].

However, some limitations of this study need to be considered. Due to its cross-sectional design, psychosocial work stress and symptoms of impaired mental health (depression and aggressiveness) were measured at the same time, and causal associations cannot be established.

Regarding the measurement instruments used, it must be noted that the PBQ [4] was originally developed for the postpartum period, conceptualized as the time period from delivery to six months after [63]. It has often been used in parent-child bonding research for this age range [64, 65]. The present study included children who were between 0–36 months of age. Therefore, it is possible that there is a measurement bias since parent-child bonding decreases with increasing age of the child. Future research should consider alternative parent-child bonding questionnaires in studies with children older than six months.

Stratified analysis for mothers and fathers regarding work stress and parent-child bonding may not have yielded statistically significant results due to an insufficient sample size. Only when effect sizes were stronger, did we find statistically significant associations for stratified analyses.

The current community sample comprised mothers and fathers who came from a rather educated and established background. The information evenings in the birth centers, which may have been attended predominantly by first-time parents, were conducted in German and the questionnaires were completed in German. Most of the participants were therefore German-speaking, which may have led to selection bias. The average of the mental health and stress factors indicated a rather healthy sample. Therefore, the results should not be generalized to families that cope with severe mental distress or that live in vulnerable, low-income family settings. It may be important to point out that most mothers with an index child under 12 months were excluded due to lack of employment during their parental leave. Hence, the distribution of mothers and fathers who had a very young child differs and the groups may not be well comparable concerning work stress variable and bonding to their child.

### Implications

Our results provide a number of implications for future research. For example, future studies should include working hours and home office as primary predictors as these appear to be significantly related to parent-child bonding. Furthermore, studies should include other mental health factors to find possible further mediation effects. In this context, the COVID-19 pandemic also poses a challenge, as reviews showed that anxiety has increased in the population [66].

Work stress can have a detrimental effect on employees’ inner stability and emotional state. Employers should ensure that employees experience the best possible balance between effort and fair compensation and appreciation for their work contributions. Especially during the COVID-19 pandemic it is critical for parents that companies expand their family-friendly features, which can alleviate stress among employees with children, e.g., offering flexible working hours and childcare options.

The study also indicated that paid work outside the home can have a highly health-promoting effect for women in general and for mothers in particular. To promote the health of all family members, it should be worthwhile to make parents and employers aware of this important opportunity.

## Conclusion

The present study detected an association between psychosocial work stress and parent-child bonding in the context of the COVID-19 pandemic after the inclusion of confounders, indicating the more psychosocial work stress, the weaker the parent-child bonding. Furthermore, the study revealed that symptoms of impaired mental health, i.e., depression and aggressiveness, played a mediating role for the association of psychosocial work stress and parent-child bonding for both parents. The particular occupational and family stress experienced by parents during the COVID-19 pandemic and the increased risk for tense and distressed behavior toward family members that may accompany it should be considered not only in research but also in the context of working conditions. In this way, both prevention as well as intervention measures related to psychosocial work stress could strengthen parent-child bonding and families. The findings need to be replicated with more diverse samples and additional mental health variables to expand on the work-family interface.

## Data Availability

The dataset analyzed during the current study is not publicly available due to legal and ethical constraints. Public sharing of participant data was not included in the informed consent of the study. All enquiries about access to data should be sent to the corresponding author. All requests to access data will be handled in accordance with the Ethics Committee of the Faculty of Medicine of the Technische Universität Dresden.
